# Genetic Variation of *COLEC10* and *COLEC11* and Association with Serum Levels of Collectin Liver 1 (CL-L1) and Collectin Kidney 1 (CL-K1)

**DOI:** 10.1371/journal.pone.0114883

**Published:** 2015-02-24

**Authors:** Rafael Bayarri-Olmos, Soren Hansen, Maiken Lumby Henriksen, Line Storm, Steffen Thiel, Peter Garred, Lea Munthe-Fog

**Affiliations:** 1 Laboratory of Molecular Medicine, Department of Clinical Immunology, Section 7631, Rigshospitalet, Faculty of Health and Medical Sciences, University of Copenhagen, Copenhagen, Denmark; 2 Department of Cancer and Inflammation Research, Institute of Molecular Medicine, University of Southern Denmark, Odense, Denmark; 3 Department of Biomedicine, Faculty of Health Sciences, Aarhus University, Aarhus, Denmark; The Hospital for Sick Children and The University of Toronto, CANADA

## Abstract

Collectin liver 1 (CL-L1, alias CL-10) and collectin kidney 1 (CL-K1, alias CL-11), encoded by the *COLEC10* and *COLEC11* genes, respectively, are highly homologous soluble pattern recognition molecules in the lectin pathway of complement. These proteins may be involved in anti-microbial activity and in tissue development as mutations in *COLEC11* are one of the causes of the developmental defect syndrome 3MC. We studied variations in *COLEC10* and *COLEC11*, the impact on serum concentration and to what extent CL-L1 and CL-K1 serum concentrations are correlated. We sequenced the promoter regions, exons and exon-intron boundaries of *COLEC10* and *COLEC11* in samples from Danish Caucasians and measured the corresponding serum levels of CL-L1 and CL-K1. The median concentration of CL-L1 and CL-K1 was 1.87 μg/ml (1.00–4.14 μg/ml) and 0.32 μg/ml (0.11–0.69 μg/ml), respectively. The level of CL-L1 strongly correlated with CL-K1 (ρ = 0.7405, P <0.0001). Both genes were highly conserved with the majority of variations in the non-coding regions. Three non-synonymous variations were tested: *COLEC10* Glu78Asp (rs150828850, minor allele frequency (MAF): 0.003), *COLEC10* Arg125Trp (rs149331285, MAF: 0.007) and *COLEC11* His219Arg (rs7567833, MAF: 0.033). Carriers of *COLEC10* Arg125Trp had increased CL-L1 serum levels (P = 0.0478), whereas promoter polymorphism *COLEC11-9570C>T* (rs3820897) was associated with decreased levels of CL-K1 (P = 0.044). In conclusion, *COLEC10* and *COLEC11* are highly conserved, which may reflect biological importance of CL-L1 and CL-K1. Moreover, the strong inter individual correlation between the two proteins suggests that a major proportion are found as heterooligomers or subjected to the same regulatory mechanisms.

## Introduction

Collectins are C-type lectins, a family of pattern recognition molecules involved in innate immunity. Collectins share a common multimeric structure where a basic subunit composed of three polypeptide chains undergoes varying degrees of oligomerization [1]. The polypeptide chains are composed of a collagen-like region, an alpha helical neck domain and a carbohydrate recognition domain (CRD). Through the clustering of the CRDs the collectins are capable of recognizing and binding to glycoconjugates on the surface of pathogens or to altered host cells, thereby facilitating their clearance [2]. Well-characterized collectins comprise mannose-binding lectin (MBL), surfactant protein A (SP-A) and D (SP-D), whose functions have been extensively studied [2]. MBL is a recognition molecule in the lectin pathway of complement along with Ficolin-1, Ficolin-2 and Ficolin-3. More recently described collectins comprises CL-L1 (alias collectin-10, collectin liver 1 or CL-10) [3], CL-K1 (alias collectin-11, collectin kidney 1 or CL-11) [4], and CL-P1 (alias collectin-12, collectin placenta 1, CL-12 or SRCL). Both CL-L1 and CL-K1 are soluble molecules that have been found circulating in blood in complex with lectin complement pathway associated serine proteases (MASPs) [5–8]. Two genes, *MASP1* and *MASP2*, encode MASP-1/-3 (alias *MASP1* isoform 1 and 2) and MASP-2 (alias MASP2 isoform 1), respectively, and encode also the alternatively spliced non-catalytic products, MAP-1 (alias MAp44 or MASP1 isoform 3) and sMAP (alias MAp19, MAP-2 or MASP2 isoform 2), respectively [9].

**Fig 1:**
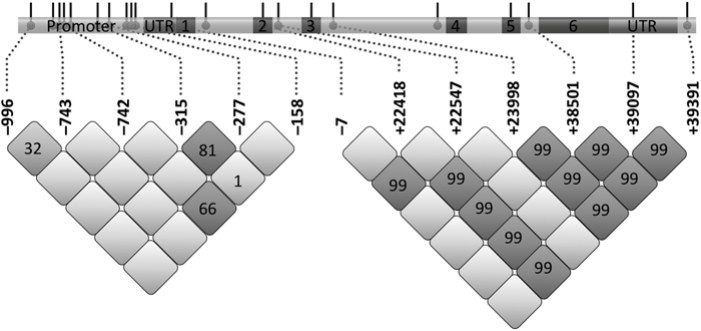
Distribution of polymorphisms and pair wise LD analysis (r^2^) observed in *COLEC10*. A schematic drawing of the gene *COLEC10* is given at the top. Exons are numbered and promoter and non-translated regions are indicated. The numbers in the grid refer to the r^2^ values of a given pair of SNPs. Darker colours of the boxes means higher r^2^ values.

The *COLEC10* gene encoding CL-L1 comprises six exons, is located on chromosome 8q23-q24.1 and is primarily expressed in the liver, placenta, and adrenal glands [3]. The protein-coding transcript gives rise to a 277 amino acid long protein with four defined regions: N-terminal segment (19 aa), collagen-like region (72 aa), alpha-helical coiled-coil neck region (34 aa), and the CRD (125 aa). The N-terminal segment and the first Gly-Xaa-Yaa repeat of the collagen-like region are encoded by exon 1 [10]; the rest of the collagen-like region by exons 2–4 (see also Fig. 1). Exon 5 encodes the neck region and exon 6 the CRD. Trimer assembly is stabilized by non-covalent interactions in the collagen-like and neck region, and inter-chain disulphide bonds between cysteine residues in the N-terminal segment (Cys12) and neck region (Cys119, Cys121) [7].

The *COLEC11* gene encoding CL-K1 comprises seven exons and is located on chromosome 2p25.3. The protein has a domain organization identical with CL-L1. CL-K1 has been found forming oligomers ranging from monomers to hexamers of the trimeric subunit, stabilized by inter chain disulfide bonds [5;8]. *COLEC11* is predominantly expressed in the liver, fetal liver, adrenals gland, small intestine, thymus, spinal cord, placenta, pancreas and kidney but low expression levels are found in many other tissues [4].

The median circulating levels of CL-L1 and CL-K1 have been estimated to be 3 and 0.3 μg/ml respectively [5;11;7]. Recently, it was shown that CL-L1 and CL-K1 form heterooligomers, and that the heterooligomers comprise a major proportion of all circulating CL-L1 and CL-K1 [8]. This feature bears resemblance with C1q, the recognition molecule of the classical pathway of complement and with other C1q-related proteins [12]. The heteromeric trimers of C1q have a composition similar to CL-L1, CL-K1 heterooligomers, with heteromeric trimers consisting of polypeptide chains originating from different genes. Formation of the collectin heterooligomers leads, in comparison with CL-L1 and CL-K1 homooligomers alone, to a substantial higher degree of oligomerization and complement activation via interaction with MASP-2 [7;8].

Carbohydrate inhibition studies have shown that CL-K1 in addition to binding to mannose-like carbohydrates (e.g. L-fucose, alpha-D-methyl-mannose and D-mannose) also bind to bacterial and yeast extracts, as well as to apoptotic cells and intact microorganisms [4;5]. Moreover, murine CL-K1 was observed to reduce influenza virus A infectivity and to bind to DNA and heparin [5;8]. Native CL-L1 present in serum showed binding to L-fucose, D-mannose but these observations may be influenced by heteromeric complex formation with CL-K1 [7].

Recently it was shown that mutations in *COLEC11* or in *MASP1* are the common underlying causes of the Carnevale, Mingarelli, Malpuech and Michels syndromes, united in the syndrome now termed 3MC [13–17]. The clinical effect of variations in the serum concentration of CL-L1 and CL-K1 is more or less unknown. However, elevated CL-K1 plasma levels have been associated with the presence of disseminated intravascular coagulation (DIC), suggesting a possible pathophysiological role for CL-K1 in uncontrolled clotting and bleeding [18].

In the present report we have studied to what extent variations in the *COLEC10* and *COLEC11* may exist and how these variations affect the circulating levels of CL-L1 and CL-K1 in healthy individuals. To provide new insight into the presence of heterooligomeres in circulation we also aimed to evaluate the degree of correlation between serum levels of CL-L1 and CL-K1.

## Materials and Methods

### Donor samples

DNA was isolated from 296 unrelated Danish Caucasian blood donors using King Fisher (Thermo Scientific). Serum from 96 of the 296 blood donors was obtained for quantification of levels of CL-L1 and CL-K1. Samples were obtained with written informed consent, and the study was approved by the Regional Ethical Committee of the Capital Region of Denmark (H2–2011–133).

### Sequencing of *COLEC10* and *COLEC11*

*COLEC10* and *COLEC11* promoters and exonic regions were amplified using PCR (for sequences of the primers see Table 1). Promoters were sequenced as three partially overlapping fragments spanning 1000 bp upstream the translation start site ATG. All forward primers included a 5′-T7 sequence (5′-TAATACGACTCACTATAGGG-3′). PCR amplifications were carried out in 12 μl volumes containing 50 ng of genomic DNA, 0.25 μM of each primer, 2.5 mM MgCl_2_, 0.2 mM dNTP, 50 mM KCl, 10 mM Tris-HCl, pH 8.4, and 0.4 units of Platinum Taq DNA Polymerase (Invitrogen). The PCR reactions were performed according to the following setup: 120s94°C, 35x(10s94°C; 30s60°C; 45s72°C), 120s72°C. The PCR products were sequenced using a T7 biotinylated primer with the ABI BigDye cycle sequencing terminator kit (Applied Biosystems). Sequencing products were purified on the PyroMark Vacuum Prep Workstation (Biotage) using streptavidin beads (Genovision), and sequence analysis was performed on an ABI Prism 3100 Genetic Analyser (Applied Biosystems). The base-called sequences were aligned using BioEdit (http://www.mbio.ncsu.edu/BioEdit/bioedit.html) and Chromas Lite (http://technelysium.com.au/) software. All chromatograms were confirmed visually.

**Table 1:** *COLEC10* and *COLEC11* primer sets.

	Forward primer	Reverse primer	Internal primer
COLEC10			
Promoter region			
Fragment 1	5′-cctttcacctaactcttagttga-3′	5′-gcctttctggtacactatctc-3′	
Fragment 2	5′-ttcgcctttctgccacatcc-3′	5′-gaactccagtgtgcatgtcag-3′	
Fragment 3	5′-tcaagtactcagcagaaggca-3′	5′-caaagccattcattgctgtgg-3′	
Exonic regions			
Exon 1	5′-ggtccatttggagcactgag-3′	5′-ataagtaacagaccagatctgg-3′	
Exon 2	5′-aagtgcatgtgaacatacatcc-3′	5′-atgcaacttgagaaacatatgag-3′	
Exon 3	5′-tagtatatattcatactcactcac-3′	5′-ccaccatgcccagctaattc-3′	
Exon 4	5′-ttatgactcatttaattagttgcac-3′	5′-actgccagagttttctcttgg-3′	
Exon 5	5′-tttagaattgtcttatagttcattc-3′	5′-cagacttgaggtgtcctagg-3′	
Exon 6	5′-tctgtgactacctcataatagg-3′	5′-tgtaacctggcgagttattgg-3′	
	5′-gagtgccatcttaccatgtac-3′	5′-aaggagaacttccaagtatagc-3′	
Pyrosequencing			
–7C>T	5′-tgtgtgttccaaatacttccc-3′	5′-[B]gactctgaatttgcaaaagaaatag-3′	5′-tgggagacccttttctgagg-3′
Glu78Asp	5′-acattagccacatttcaaggga-3′	5′-[B]acctcggtctctgagtgttg-3′	5′-gtttgcttttcaggaattaaagg-3′
Arg125Trp	5′-[B]cgtgtcttcatcggcatca-3′	5′-ctccttgtcaaactcacacatga-3′	5′-ccacttgttgaaggtccgca-3′
COLEC11			
Promoter region			
Fragment 1	5′-tgtctggttttggtatcagggt-3′	5′-gcttctatgcagcccgaatc-3′	
Fragment 2	5′-tcacctttgaacctagcttctta-3′	5′-cctggggagtgtgggaac-3′	
Fragment 3	5′-ttgggatgttcgggttggag-3′	5′-atcaaactttggaactctggtg-3′	
Exonic regions			
Exon 1	5′-ttgggatgttcgggttggag-3′	5′-atcaaactttggaactctggtg-3′	
Exon 2	5′-ttggtgcctggtgctgacc-3′	5′-ggaattccatggccgaagac-3′	
Exon 3	5′-aaacacaggccctttctaagag-3′	5′-ccacaggtcagcacaaggag-3′	
Exon 4	5′-ctccattctatagtgtgtatgac-3′	5′-agctgcagcccctccatcc-3′	
Exon 5	5′-aaagaatgatcactcgataatcc-3′	5′-gtgtccaccgtgggatgtg-3′	
Exon 6	5′-ttgaaatacatgtgtctgccct-3′	5′-gcacagccacaggcagcag-3′	
Exon 7	5′-acctcccagccctgtcctg-3′	5′-cattttctcagtttagacaacagc-3′	
	5′-cccaacaatgcctacgacg-3′	5′-catggataatagtgtgaaggaacc-3′	
Pyrosequencing			
His219Arg	5′-[B]tggaaagagagcaaatgttcct-3′	5′-actggaattgtagtgttgatgga-3′	5′-actaatatccagttgtccaacaaa-3′

Forward and reverse primers were used for PCR amplification, internal primer was used for pyrosequencing. All forward primers for the promoter and exonic regions include a 5′-T7 sequence (5′-TAATACGACTCACTATAGGG-3′). Biotinylated oligos are indicated as [B].

### Pyrosequencing-based genotyping assay

Pyrosequencing was carried out as described by Munthe-Fog *et al*. [19]. In brief, specific primer sets were designed to amplify the three non synonymous SNPs found in *COLEC10* and *COLEC11*, (rs150828850, rs149331285 and rs7567833) and the SNP located in the 5′UTR of *COLEC10* (ss749616235) (for primer list see Table 1). DNA was PCR amplified and purified as described above. Purified PCR products were incubated 5 min at 85°C with 10 μM of internal primer and annealing buffer (magnesium acetate 2 mM, Tris 20 mM, pH 7.6). The pyrosequencing reaction and sequence analysis were carried out in the PSQ 96MA (Biotage).

### SNP identification

The observed polymorphisms (Tables 2 and 3) can be found in NCBI’s SNP database (http://www.ncbi.nlm.nih.gov/projects/SNP/) through accession numbers (rs) or submission numbers (ss).

**Table 2:** Genetic variation observed in the *COLEC10* gene.

Variation	dbSNP	Region	Amino acid change	AA	Aa	aa	MAF
−996C>T	rs117863403	Promoter	-	93 (96.9%)	3 (3.1%)	0	0.016
−796delT	ss991382312	Promoter	-	95 (99%)	1 (1%)	0	0.005
−743C>T	ss991382313	Promoter	-	95 (99%)	1 (1%)	0	0.005
−742G>A	rs3812490	Promoter	-	94 (98%)	2 (2%)	0	0.010
−663C>G	rs1485297	Promoter	-	81(84.4%)	14 (14.6%)	1 (1%)	0.083
−315G>C	rs1485298	Promoter	-	62 (64.6%)	28 (29.2%)	6 (6.3%)	0.208
−277T>C	rs2450048	Promoter	-	56 (58.3%)	33 (34.4%)	7 (7.3%)	0.245
−161_−157 delAAAAT	rs148350292	Promoter	-	95 (99%)	1 (1%)	0	0.005
−150C>T	rs3829048	Promoter	-	93 (96.9%)	3 (3.1%)	0	0.016
−145G>A	ss991382317	Promoter	-	95 (99%)	1 (1%)	0	0.005
−7C>T	ss749616235	5′ UTR	-	299 (99.7%)	1 (0.3%)	0	0.005
+228A>G	rs2465383	Intron 1	-	26 (26%)	55 (55%)	19 (19%)	0.465
+22418A>G	rs16891987	Exon 2	-	97 (97%)	3 (3%)	0	0.015
+22547T>C	rs149290883	Intron 2	-	99 (99%)	1 (1%)	0	0.005
+23881A>C	rs150828850	Exon 3	Glu78Asp	298 (99.3%)	2 (0.7%)	0	0.005
+23998A>T	rs4512407	Intron 3	-	97 (97%)	3 (3%)	0	0.015
+36545C>T	rs149331285	Exon 5	Arg125Trp	296 (98.7%)	4 (1.3%)	0	0.005
+38501C>A	rs11987106	Intron 5	-	97 (97%)	3 (3%)	0	0.015
+39097A>G	rs1064556	3′ UTR	-	97 (97%)	3 (3%)	0	0.015
+39391C>G	rs1064557	3′ downstream	-	97 (97%)	3 (3%)	0	0.015

The base position refers to the location of the polymorphism considering the A from the translation start site ATG as +1, and the adjacent 5′ base as -1. All variations adhered to the Hardy-Weinberg equilibrium. ‘AA’, individuals homozygote for the major allele; ‘Aa’, heterozygous individuals; ‘aa’, individuals homozygous for the minor allele; MAF, minor allele frequency.

**Table 3:** Genetic variation observed in the *COLEC11* gene.

Base position	dbSNP	Region	Amino acid change	AA	Aa	aa	MAF
−10007_− 10004delCATT	ss991382311	Promoter	-	95 (99%)	1 (1%)	0	0.005
−9970T>C	rs1864480	Promoter	-	53 (55.2%)	40 (41.7%)	3 (3%)	0.240
−9766T>C	rs4849953	Promoter	-	44 (45.8%)	44 (45.8%)	8 (8.4%)	0.313
−9591G>T	rs34596301	Promoter	-	86 (89.6%)	10 (10.4%)	0	0.052
−9570C>T	rs3820897	Promoter	-	52 (54.2%)	41 (42.7%)	3 (3%)	0.245
−9175T>C	rs77246730	5′ UTR	-	193 (98.5%)	3 (1.5%)	0	0.010
+8822C>T	rs3811531	Intron 2	-	36 (36%)	46 (46%)	18 (18%)	0.410
+8926G>A	rs7567724	Intron 2	-	77 (77%)	20 (20%)	3 (3%)	0.130
+8939G>A	rs17017752	Intron 2	-	95 (95%)	4 (4%)	1 (1%)	0.030
+9072G>A	rs67826307	Intron 3	-	95 (95%)	4 (4%)	1 (1%)	0.030
+9091C>T	rs72769325	Intron 3	-	97 (97%)	3 (3%)	0	0.015
+33064T>G	ss749616236	Intron 3	-	99 (99%)	1 (1%)	0	0.005
+33116C>T	rs3811528	Intron 3	-	49 (49%)	40 (40%)	11 (11%)	0.310
+33119G>T	rs36024497	Intron 3	-	99 (99%)	1 (1%)	0	0.005
+33125G>A	ss749616237	Intron 3	-	99 (99%)	1 (1%)	0	0.005
+33144A>G	rs3811527	Intron 3	-	49 (49%)	40 (40%)	11 (11%)	0.310
+33145C>T	rs148763047	Intron 3	-	98 (98%)	2 (2%)	0	0.010
+33233T>C	rs34347318	Exon 4	-	98 (98%)	2 (2%)	0	0.010
+33245T>C	rs17017791	Exon 4	-	99 (99%)	1 (1%)	0	0005
+35844C>T	rs17017804	Intron 4	-	99 (99%)	1 (1%)	0	0.005
+35846C>T	rs144784212	Intron 4	-	99 (99%)	1 (1%)	0	0.005
+39141C>T	rs34436491	Exon 6	-	99 (99%)	1 (1%)	0	0.005
+39618C>G	rs7567833	Exon 7	His219Arg	280 (93.3%)	20 (6.7%)	0	0.025
+39739C>T	rs114716171	Exon 7	-	96 (96%)	4 (4%)	0	0.020

The base position refers to the location of the polymorphism considering the A from the translation start site ATG as +1, and the adjacent 5′ base as -1. All variations adhered to the Hardy-Weinberg equilibrium. ‘AA’; individuals homozygote for the major allele, ‘Aa’; heterozygous individuals, ‘aa’; individuals homozygous for the minor allele; MAF, minor allele frequency.

### *In silico* prediction of the biological consequences of variations

*In silico* analysis of functional effects was performed on the observed nsSNPs. PolyPhen-2 (http://genetics.bwh.harvard.edu/pph2/) evaluates physical properties, proximity to functional structures, and the evolutive conservation to predict the functional significance of a mutation using a trained Naïve Bayes classifier [20]. BLOSUM62 (http://www.ncbi.nlm.nih.gov) is an amino acid substitution matrix that assigns a score to each aligned pair of residues based on the odds of finding both amino acids in an alignment on purpose rather than by chance. SIFT (http://sift.bii.a-star.edu.sg/) is a multi-step sequence alignment comparison algorithm that estimates whether an amino acid substitution may have an effect on protein function, based upon the premise that highly conserved residues are more intolerant to substitution than those less conserved [21;22]. Align-GVGD (http://agvgd.iarc.fr/) classifies a substitution from most likely to least likely to interfere with protein function based on multiple sequence alignments (MSA) and the combined Grantham Variation (GV) and Grantham Deviation (GV) scores, which measure the biochemical distance between aminoacids [23]. Protein MSA was constructed using ClustalW included in the program Bioedit (www.mbio.ncsu.edu/BioEdit/bioedit.html). CL-L1 and CL-K1 protein sequences were aligned with their orthologues from 17 different species in order to build a dataset with a sufficient size and alignment depth to compensate for the appearance of constrained positions due to chance [24;25]. PhD-SNP is a trained support vector machine-based predictor that classifies mutations as neutral polymorphisms or disease related based on sequence and profile information [26]. Furthermore, an evolutionary conservation profile was generated by Consurf [27]. This web-based program calculates the degree of conservation (from most variable to most conserved) for every position of the protein using MSA and an empirical Bayesian algorithm.

### CL-L1 and CL-K1 levels in serum

The serum levels of CL-L1 and CL-K1 was quantified using validated double sandwich immuno assays as previously described in detail [7] and [28].

### Statistical analysis

Hardy-Weinberg was calculated applying the χ^2^ test to the simple gene counting results, implementing Yates’ correction when considered appropriate. Linkage disequilibrium (LD), expressed as r^2^, and observed haplotypes were assessed by SHEsis software [29;30]. The statistical significance of the genetic impact on serum levels was tested with Mann-Whitney U test, Kruskal-Wallis test and Spearman Rank correlation. Whiskers and outliers were calculated according to Tukey’s method.

## Results

### Genetic variation in *COLEC10* and *COLEC11*

We sequenced the promoter region spanning 1000 bp upstream the translation start site, the exon and intron/exon boundaries in *COLEC10* and *COLEC11*.

***COLEC10***. A total of 10 known and novel variations were observed in the promoter region (Table 2) including a novel variation located in the 5’UTR. Another 10 variations were observed in the exons and the flanking regions. Two low frequent variations (MAF < 0.01) resulted in an amino acid change: Glu78Asp (rs150828850) and Arg125Trp (rs149331285). The Glu78Asp variation in exon 3 occurs in a variable residue and was predicted to be benign, in contrast to Arg125Trp in exon 6, which is located in an evolutionary constrained position in the neck domain and was by *in silico* analysis predicted to be potentially critical for the structure (Table 4). A complete list of observed variations and linkages are given in Table 2 and Fig. 1. To test whether any of the identified variations were located within a regulatory domain, all promoter variations were analyzed *in silico* (SwissRegulon Database). *COLEC10 161_-157AAAATdel* overlaps with a SRY and several Forkhead box (FOX) binding sites. SRY and the FOX family of transcription factors are known regulators of multiple cellular and developmental processes such as liver differentiation [31;32]. Of interest, additional perfect LD was observed between seven loci; *COLEC10–7* (ss749616235), *+22418* (rs16891987), *+22547* (rs149290883), *+23998* (rs4512407), *+38501* (rs11987106), *+39097* (rs1064556), and *+39391* (rs1064557), however, these minor alleles were clustered in 4 individuals only.

**Table 4:** Non-synonymous variations in *COLEC10* and *COLEC11*.

Variation	Region	PolyPhen-2	Blosum62	SIFT	Align-GVGD	PhD-SNP	Consurf
*COLEC10* Glu78Asp (rs150828850)	Exon 3	Benign	2	1	C0	Neutral (7)	Variable(3)
*COLEC10* Arg125Trp (rs149331285)	Exon 5	Probably damaging	-3	0	C65	Disease (3)	Conserved (9)
*COLEC11* His219Arg (rs7567833)	Exon 7	Benign	0	0.462	29	Neutral (3)	Variable(3)

PolyPhen-2 classifies qualitatively a mutation as benign, possibly damaging, or probably damaging. BLOSUM62 matrix values range from 4 (more likely) to -4 (less likely substitutions). SIFT values >0.05 indicate substitutions with little effect on protein function, while values <0.05 point out possible deleterious substitutions. Align-GVGD categorizes a mutation in seven graded classifiers from less likely (class C0) to most likely to interfere with protein function (class C65). PhD-SNP produces a binary prediction (neutral vs disease-related polymorphism), together with the reliability score of the prediction (0–9). Consurf position-specific conservation scores are divided in 9 grades, from the most variable (grade 1) to the most conserved positions (grade 9).

**Fig 2:**
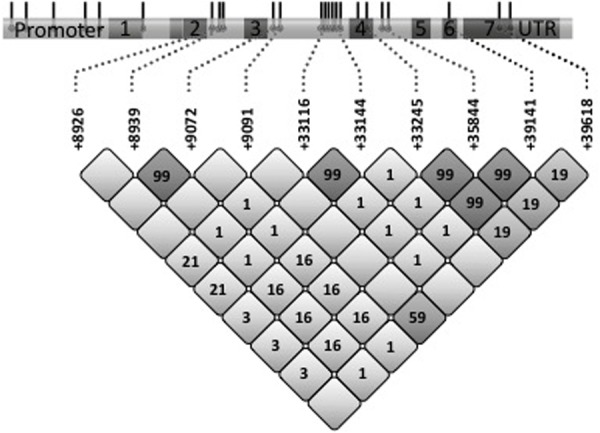
Distribution of polymorphisms and pair wise LD analysis (r^2^) observed in *COLEC11*. A schematic drawing of the gene *COLEC11* is given at the top. Exons are numbered and promoter and non-translated regions are indicated. The numbers in the grid refer to the r^2^ values of a given pair of SNPs. Darker colours of the boxes means higher r^2^ values.

***COLEC11***. Four known variations and a novel 4-bp deletion were observed in the promoter region (Fig. 2, Table 3). In the 5’UTR and the intron/exon boundaries 12 known and two novel variations were identified. Further five variations were found within the exons, one resulting in an amino acid change: His219Arg (rs7567833) located in exon 7, which encodes the carbohydrate recognition domain of CL-K1. Only individuals heterozygous for His219Arg were identified (N = 5, MAF 0.025). His219Arg showed a moderate linkage with *+33245* in exon 4 (rs17017791), *+35844* in intron 4 (rs17017804), *+39141* in exon 4 (rs34436491); and more robust linkage with *+9091* in intron 3 (rs72769325). In addition, two SNPs in intron 3, rs3811528 and rs3811527 (*+8939* and *+9072*), with MAFs of 0.31 had a perfect LD.

### Serum levels

**Fig 3:**
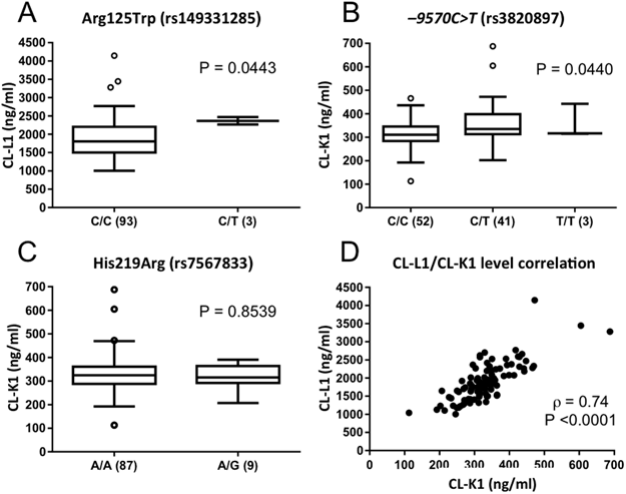
Impact of allelic variants on serum levels. A-C, the serum levels of CL-L1 and CL-K1 and the corresponding genotype. Horizontal bars indicate median values; number in parenthesis, number of individuals and white circles, outliers. Statistical significance was determined by non-parametric Mann-Whitney and Kruskal-Wallis tests. D, the distribution of CL-L1 and CL-K1 levels in the cohort. The strength of the correlation was calculated using Spearman Rank.

CL-L1 mean serum concentration was estimated to be 1.87 μg/ml (range 1.00—l4.14 μg/ml). CL-K1 serum concentration ranged from 0.11 to 0.69 μg/ml, with a mean concentration of 0.32 μg/ml. None of the highly frequent polymorphisms in the *COLEC10* promoter region were associated with CL-L1 serum levels. Interestingly, although no individuals homozygous for the minor allele were found, individuals heterozygous for Arg125Trp (rs149331285) (N = 3) had significantly higher levels of CL-L1 in serum (P = 0.0478, Fig. 3A).

In *COLEC11* the promoter polymorphism—*9570C>T* (rs3820897) was significantly associated with higher levels of CL-K1 (P = 0.044, Fig. 3B). The non-synonymous SNP; His219Arg had no significant effect on the level of CL-K1 (Fig. 3C). The allele count for several of the genotyped positions was too low to significantly determine the impact on the level of circulating proteins. A strong correlation, independent of age and gender (not shown) between CL-L1 and CL-K1 (ρ = 0.7405, P <0.0001) was observed (Fig. 3D). However, none of the identified genetic variations in *COLEC10* were significantly associated with the circulating levels of CL-K1 and vice versa (data not shown), thus *COLEC10* Arg125Trp was not associated with the level of CL-K1 (P = 0.916) and neither was *COLEC11–9570C>T* with the level of CL-L1 (P = 0.938).

## Discussion

To study how the genetic variation affects structure and concentrations in the background population we examined the *COLEC10* and *COLEC11* in healthy individuals and determined the circulating levels of CL-L1 and CL-K1 in corresponding serum samples.

Screening *COLEC10* and *COLEC11*, we found two variations with a putative impact on either serum level and/or protein structure. *COLEC10* Arg125Trp (rs149331285) was predicted by *in silico* analysis to have a significant effect on protein structure. This nsSNP is located in the neck domain, an alpha helical coiled-coil region involved in the trimerization of the collagen chains. The helical coil region consists of three parallel right-handed alpha helices with hydrophobic residues repeated in a heptad pattern. The association of the alpha helices aligns the collagen chains allowing them to fold in a zipper-like fashion. There is extensive evidence that oligomerization of SP-D monomers is required for high-affinity binding to carbohydrates [33] and for many of its biological functions [34;35]. Thus, *COLEC10* Arg125Trp may be of considerable importance if the substitution disrupts the coil structure causing the trimers to fall apart or affect the assembly of heteromeric complexes. Curiously, the level of CL-L1 was significantly higher in the three heterozygous individuals we identified. With a calculated MAF of 0.005, this variation would be interesting to characterize further.

Although only present in three individuals the promoter polymorphism *COLEC11–9570C>T* (rs3820897) was significantly associated with the level of CL-K1 (P = 0.044). We identified several other variations that potentially could influence the protein levels in serum. However, due to limitations in our cohort size the impact of the genetic variation on the circulating levels require further analyses in larger cohorts. Among the more interesting variations is the *COLEC10–161–157AAAATdel* expected to interrupt the binding site of several transcription factors that regulate an array of events ranging from liver development (e.g. FOXA2) [31] to immune response modulation (FOXJ1, FOXO3, FOXQ1) [36]. Additional studies into *COLEC10* and *COLEC11* regulatory cassettes could resolve these questions.

No effect on serum levels was observed for the polymorphism *COLEC11* His219Arg (rs7567833, MAP 0.033), located in the carbohydrate recognition domain. Phylogenetic analysis revealed that the *G* allele (the minor allele) is indeed the ancestral allele. Although located adjacent to the ligand binding site in the CRD, this substitution could affect the binding affinity of the variant molecule towards its ligand as seen for Ficolin-2 [37]. Amino acid substitutions in the pathogen recognition domain affecting the ligand binding have previously been reported for Ficolin-2. Two non-synonymous polymorphisms in *FCN2* positioned near the binding site markedly alter the binding capacity for GlcNAc and thus the complement activation potential [37]. Several Ficolin-2 clinical associations have been reported and genotypes conferring low lectin activity have been associated with increased risk of infections [38]. Thus, *COLEC11* His219Arg could be of clinical relevance.

Recently, heteromeric complexes between CL-L1 and CL-K1, stabilized by disulfide bonds, were observed in circulation by Henriksen *et al* [8]. The observation was further supported by co-transfections of the two proteins in CHO cells in which the ratio of CL-L1 and CL-K1 in the formed heteromeric complexes was estimated to 1:2 in favor of CL-K1. Both recombinant CL-K1 and CL-L1 and the heterocomplexes from serum are able to form complexes with the MASPs leading to activation of the complement cascade. In addition, the complement activation potential of the heterocomplexes was reported to be more potent than the potential observed for CL-K1 homocomplexes [8].

In the present study we observed a strong correlation between CL-L1 and CL-K1 serum levels (ρ = 0.7405, Fig. 3C), independent of age or gender lending support to the above observation that a major proportion of CL-L1 and CL-K1 exist as heterooligomeric complexes in the circulation whereas only a minor part of CL-L1 and CL-K1 circulates as homocomplexes. A more definite prediction of the composition of the heterocomplexes i.e. the CL-L1:CL-K1 ratio would be highly speculative as the relatively large difference in levels between CL-L1 and CL-K1 could be due to differences in the techniques for estimating the protein concentration.

In conclusion, we report the finding of three gene variations in *COLEC10* and *COLEC11* with putative effect on the circulating levels and function of CL-L1 and CL-K1. *COLEC10* Arg125Trp (rs149331285) was predicted to have a significant effect on the protein structure however carriers of the variant had significant higher levels of circulating CL-L1. In *COLEC11* the promoter polymorphism *COLEC11–9570C>T* (rs3820897) was significantly associated with the level of CL-K1. Furthermore, located in the CRD of CL-K1 the polymorphism *COLEC11* His219Arg (rs7567833) could potentially affect the binding capacity of CL-K1 towards its ligand thereby altering the lectin pathway activation potential of CL-K1. Thus, our study offers an overview of *COLEC10* and *COLEC11* sequence-variant footprint in the Caucasian population and the observations made here will serve to establish more detailed footprint for genotype-phenotype studies in the Caucasian population as well as in other ethnic groups.
